# Supplementary material for: The Ser290Asn and Thr715Pro Polymorphisms of the SELP Gene Are Associated with A Lower Risk of Developing Acute Coronary Syndrome and Low Soluble P-Selectin Levels in A Mexican Population
Source: Biomolecules. 2020 Feb 11;10(2):270. doi: 10.3390/biom10020270 (PMC7072273; doi:10.3390/biom10020270)
Supplement: Supplementary file 1 [file biomolecules-10-00270-s001.pdf]

Supplementary Table S1. Distribution of Ser290Asn and Thr715Pro *SEL-P* polymorphisms in individuals with and without T2DM.

	n (genotype frequencies)			MAF	Model	OR (95%CI)	*pC*
SELP G1057A Ser290Asn (rs6131)							
With T2DM	*GG*	*GA*	*AA*				
(n=281)	243 (0.865)	37 (0.132)	1 (0.004)	0.07	Co-dominant	0.98 (0.66-1.46)	0.74
					Dominant	0.96 (0.65-1.42)	0.83
Without					Recessive	0.96 (0.06-3.84)	0.44
T2DM	867 (0.843)	152 (0.148)	9 (0.009)	0.08	Over-dominant	0.99 (0.67-1.47)	0.96
(n=1028)					Additive	0.93 (0.65-1.35)	0.72
SELP A2331C Thr715Pro (rs6136)							
With T2DM	*AA*	*AC*	*CC*				
(n=274)	233 (0.850)	38 (0.139)	3 (0.011)	0.08	Co-dominant	1.74 (0.78-1.76)	0.57
					Dominant	1.20 (0.81-1.78)	0.36
Without					Recessive	1.70 (0.43-6.39)	0.47
T2DM	885 (0.868)	126 (0.124)	8 (0.008)	0.07	Over-dominant	1.17 (0.78-1.75)	0.46
(n=1019)					Additive	1.21 (0.84-1.72)	0.31

ACS, Acute coronary syndrome; MAF, Minor allele frequency; OR, odds ratio; CI, confidence interval; pC, *p*-value. The p-values were calculated by the logistic regression analysis, and ORs were adjusted for gender, age, blood pressure, BMI, total cholesterol, HDL-C, LDL-C, triglycerides, and smoking habit.

Supplementary Table S2. Distribution of Ser290Asn and Thr715Pro *SEL-P* polymorphisms in individuals with and without hypertension.

	n (genotype frequencies)			MAF	Model	OR (95%CI)	*pC*
SELP G1057A Ser290Asn (rs6131)							
With hypertension	*GG*	*GA*	*AA*				
(n=554)	483 (0.872)	68 (0.123)	3 (0.005)	0.07	Co-dominant	0.77 (0.55-1.07)	0.25
					Dominant	0.76 (0.55-1.06)	0.10
Without					Recessive	0.65 (0.16-2.60)	0.53
hypertension	627 (0.830)	121 (0.160)	7 (0.009)	0.09	Over-dominant	0.78 (0.56-1.08)	0.13
(n=755)					Additive	0.78 (0.57-1.05)	0.19
SELP A2331C Thr715Pro (rs6136)							
With hypertension	*AA*	*AC*	*CC*				
(n=545)	480 (0.881)	63 (0.116)	2 (0.004)	0.06	Co-dominant	0.80 (0.56-1.14)	0.13
					Dominant	0.76 (0.54-1.07)	0.12
Without					Recessive	0.32 (0.07-1.54)	0.12
hypertension	638 (0.853)	101 (0.135)	9 (0.012)	0.08	Over-dominant	0.81 (0.57-1.15)	0.23
(n=748)					Additive	0.75 (0.55-1.3)	0.14

ACS, Acute coronary syndrome; MAF, Minor allele frequency; OR, odds ratio; CI, confidence interval; pC, *p*-value. The p-values were calculated by the logistic regression analysis, and ORs were adjusted for gender, age, BMI, total cholesterol, HDL-C, LDL-C, triglycerides, and smoking habit.
